# Current Trends in Facelift and Necklift Procedures

**DOI:** 10.3390/jcm14124273

**Published:** 2025-06-16

**Authors:** Carter J. Boyd, Daniel J. Ceradini

**Affiliations:** Hansjörg Wyss Department of Plastic Surgery, NYU Langone, New York, NY 10016, USA

**Keywords:** facelift, necklift, rhytidectomy, deep-plane facelift, deep-plane necklift, facial anatomy, facial rejuvenation

## Abstract

Many surgical and nonsurgical options are available to patients seeking facial rejuvenation. In this review, we aim to provide an overview of the current trends in facelift and necklift procedures while simultaneously highlighting the utility of nonsurgical treatments. A comprehensive literature review was performed using the PubMed, Google Scholar, and Cochrane Library databases, with the objective of including recent literature published on facelift and necklift procedures from 2015 to 2025. Articles were selected based on relevance, with a specific focus on including a wide breadth of techniques. A considerable body of literature has been published to further classify the soft-tissue anatomy of the face and neck. In particular, these studies focus on the characterization of the three-dimensional anatomy of the facial nerve with emphasis on safe planes of dissection to avoid inadvertent facial nerve injury. The current literature continues to debate both the theoretical and practical advantages and disadvantages of various facelift techniques. Broadly speaking, facelift techniques can be divided into those that manipulate the superficial musculoaponeurotic system (SMAS) layer on its superficial surface and those that undermine the SMAS to varying extents. Numerous approaches are available to improve the contour of the neck and jawline, including manipulation of the platysma muscle and subplatysmal volume reduction. Other surgical procedures and nonsurgical treatments should be considered to optimize and enhance facelift and necklift results. Advancements in patient safety include a focus on minimizing complications while reducing the length of recovery. Facelift and necklift procedures remain the foundational pillars for facial rejuvenation. With attention to patient-specific anatomy, surgeons can work collaboratively with patients to provide global facial optimization by choosing appropriate facelift and necklift techniques in combination with other ancillary procedures. Doing so will deliver enduring, elegant results.

## 1. Introduction

While many trends come and go, one constant in facial rejuvenation is the timeless elegance of natural results that prompt an onlooker to question “Did they, or didn’t they?” As procedures come in and out of fashion, the plastic surgeon must focus on achieving long-lasting results that will age gracefully with a patient. While various nonsurgical procedures have emerged over the past several decades, facelift and necklift procedures remain the principal modalities for treating signs of advanced aging in the lower face, jawline, and neck [1]. In recent years, surgeons have attempted to reduce the stigmata of these procedures while enhancing the longevity of these interventions [2]. Such pursuits have reinvigorated prior debates comparing surgical techniques and introduced new innovations. These events have spawned continual evolution as surgeons seek to deliver durable results with fewer complications and abbreviated recovery [3].

A wide catalog of surgical and nonsurgical options for facial rejuvenation is available to patients aiming to achieve a more youthful appearance [4]. Given the number of available options, comprehensive facial assessment and attentive understanding of a patient’s goals are cornerstones of initial evaluation [5]. Furthermore, today’s patients are more informed than ever, as social media has facilitated direct-to-consumer marketing [6]. The patient and plastic surgeon should collaboratively determine the best surgical and/or nonsurgical approaches to provide an enduring outcome congruent with the patient’s desire [7].

In this review, we aim to provide an overview of the current trends in surgical rejuvenation of the face and neck, discuss ongoing controversies, emphasize the importance of customizing surgical approaches to individual patient anatomy, highlight complete facial assessment, introduce the utility of ancillary procedures, and discuss salient points in patient safety.

## 2. Materials and Methods

A comprehensive literature review was conducted by using multiple databases, including PubMed, Google Scholar, and Cochrane Library. The search focused on articles published up to April 2025, with specific weight placed on studies published in the past decade. Keywords included a combination of the following: “facelift”, “necklift”, “facelift anatomy”, “necklift anatomy”, “rhytidectomy”, “deep neck lift”, “deep plane facelift”, “composite facelift”, “SMAS facelift”, “bilamellar facelift”, “submandibular gland reduction”, “submandibular gland resection”, “facelift complications”, “necklift complications”, and “facial rejuvenation”. Articles were selected to sample an appropriate diversity of surgical techniques and to synthesize both recent and relevant literature in order to provide an appropriate narrative review of ongoing trends in facial rejuvenation. Topics of interest included the surgical anatomy of facelift and necklift procedures, facelift techniques, necklift techniques, patient safety, and ancillary facial rejuvenation procedures.

## 3. Discussion

### 3.1. Renewed Attention to Anatomy

Reinvigorated interest has been placed on improving the understanding of the three-dimensional anatomy of the face related to various techniques for rhytidectomy [8]. While the anatomy of the face, including the soft-tissue layers, course of the facial nerve, and retaining ligaments, has not changed, inconsistent nomenclature has confused and mystified facial anatomy [9]. Highlighting this very point, the anatomic existence of the superficial musculoaponeurotic system (SMAS) layer—a foundational principle for plastic surgeons performing facelifts that was originally described by Mitz and Peyronie—has been called into question [10]. Backed by histologic evaluation, Minelli and colleagues suggest that the SMAS does not exist anatomically, and the term rather denotes a surgically dissected flap of variable thickness that contains fat, facial mimetic muscles, and fascia [11]. While such nuances may appear academic, these inconsistencies exacerbate discrepancies in the nomenclature and obfuscate the understanding of surgical anatomy. Furthermore, they disrupt inter-surgeon discussion as the nomenclature is conflated both in everyday use and in the literature. Intimate to the discussion of the SMAS, the concentric soft-tissue layers of the head and neck have remained controversial since their earliest descriptions [12]. Traditional teaching describes concentric layers moving from superficial to deep skin, subcutaneous fat, the SMAS, space/retaining ligaments, and deep fascia/periosteum [13]. More recent developments have suggested that rather than considering the fourth layer (layer of retaining ligaments and space) as a distinct layer, this layer simply represents a potential layer in which the deep-plane dissection occurs [14]. Still others describe this same layer as a plane of loose areolar connective tissue [13]. Deep fascia has carried different meanings for surgeons and anatomists. While this has been recognized, it has not stopped the conflation of the terms. Others have inserted eponyms for the deep fascial layer, only increasing the confusion [15].

With increased attention to deep-plane and sub-SMAS techniques, many studies have focused on classifying the course of the facial nerve to aid in safe facelift dissection [14]. Stuzin and Rohrich outline key facial nerve danger zones where the facial nerve branches are more superficially positioned, making them susceptible to injury during dissection. Furthermore, they emphasize that while the two-dimensional course of the nerve is variable amongst patients, the nerve branches traverse the face at a predictable depth, which is the key to avoiding motor nerve injury [16]. Minelli and colleagues detail the depth of the facial nerve branches, describing their course from deep in the parotid gland towards the facial mimetic musculature. Importantly, they emphasize that the facial nerve branches run within the deep fascia, not under the deep fascia, and transition superficially to innervate their target musculature. At these locations, the nerve branches are at their highest risk of injury during deep-plane/sub-SMAS dissection [14]. Lindsey and associates detail the three-dimensional anatomy of the cervical and marginal mandibular branches of the facial nerve as they course through the fascial layers of the face. The authors describe the cervical line, which describes the location where the terminal branch or branches of the cervical branch emerge through the deep cervical fascia—namely at or distal to a line from a point 5 cm below the mandibular angle on the anterior border of the sternocleidomastoid muscle to the point where the facial vessels cross over the mandibular border. Considering this anatomy, the authors pose that sub-plastysmal dissection in the neck can be safely continued superiorly over the mandibular border if performed proximal to this zone [17,18,19].

Numerous reports have outlined specific surgical techniques while focusing on anatomy to guide safety in deep-plane dissections [20,21,22,23,24,25,26]. In the neck, supraplatysmal dissection is safe, devoid of risk to nerve branches [27]. Subplatysmal dissection should proceed cautiously with limited use of high levels of electrocautery laterally where the nerves are located [28]. In the face, the deep-plane/sub-SMAS entry point is determined by a line from the lateral canthus to the gonial angle, with attention paid to staying anterior to Pitanguy’s line to avoid frontal nerve injury [29]. This entry point also places the deep-plane/sub-SMAS entry point at the junction of the fixed and mobile SMAS [29]. Following sharp entry into the deep-plane/sub-SMAS layer, blunt dissection should be preferentially used to avoid unintended facial nerve branch transection [15]. Critical landmarks of this technique are the identification and release of the zygomatic ligaments (McGregor’s patch), with attention paid to staying on the superficial surface of the zygomaticus major muscle as the surgeon dissects anteriorly [30]. The deep-plane dissection extends sub-plastysmal into the neck with the release of the cervical retaining ligaments [20].

The requirement of deep-plane/sub-SMAS release of the facial retaining ligaments to achieve adequate mobility of the facial soft tissues remains controversial [31]. Minelli and associates examined 50 cadavers to further classify the consequences of various levels of surgical release on the relative mobility of the face. The authors conclude that minimal dissection techniques alter the architecture such that the supportive role and antigravitational lattice of retaining fibers are lost, leading to reduced longevity of the surgical result [31]. By contrast, they describe that undermining with release of the deep ligaments in the facial glide plane enables redraping of the overlying tissue and preserves the natural architecture. This enables repositioning of the overlying skin and soft tissues in a composite manner that maintains their structural integrity [31]. In another cadaver study, Jacono and colleagues describe significantly more movement of the facelift flap with deep-plane/sub-SMAS release of the retaining ligaments compared to plication procedures [32]. Others argue that aging leads to laxity in the retaining ligaments of the face, and sufficient mobility of the face can be achieved without the release of retaining ligaments with superficial SMAS manipulation techniques (SMAS plication, SMASectomy) [33]. The management of the mandibular ligament remains controversial. Originally described by Furnas, the mandibular ligament represents the anterior border of the jowl [34]. There are two main theories of the anatomic basis of the pathophysiology of the jowl. These include subplatysmal and supraplatysmal mechanisms [35]. Recent anatomical analysis suggests that the mandibular ligament only exists in the subplatysmal plane without extension through the subcutaneous plane [36]. Based on this understanding, Talei describes that the release of the mandibular ligament may not be required to achieve the desired improvement in this region [35].

In another cadaver study bolstered with histologic analysis, Minelli and colleagues detail the platysma in relation to the overlying skin, reporting that the platysma is adherent to both the overlying skin and deep fascia across its entire superficial and deep surface. This anatomy is particularly important when managing platysmal bands, and the authors provide anatomical explanations for failures of some platysma techniques [27]. Just as the fat compartments of the face have been well classified, further attention has been paid to understanding the fat compartments of the neck [37,38]. Addressing the deep structures of the neck, surgeons should understand the detailed anatomy of the submental and submandibular triangles [39]. The large volume of recent literature detailing anatomy highlights the complexity of the face and neck and underscores the importance of a strong anatomical foundation when approaching surgical rejuvenation of the face and neck [8]. This foundation is critical to deciphering the various techniques that can be employed and understanding the effects each can create.

### 3.2. Facelift Technique

**Figure 1:**
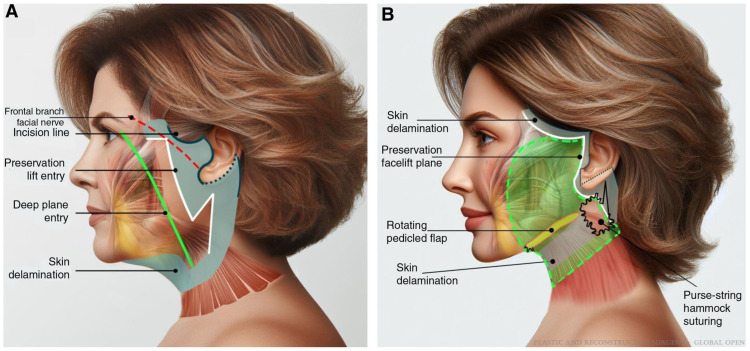
(**A**) Depiction of the standard deep-plane facelift entry point (green) and preservation facelift entry point (white). (**B**) With the preservation facelift design, there is a limited area of delaminated skin following the suspension of the superficial musculoaponeurotic system (SMAS). This figure was reused with permissions under CC-BY with Wolters Kluwer Health, Inc. (Philadelphia, PA, USA), License Number 6045410419117. Image is from Figure 2 of Sadati, Kevin Do et al. [50].

Addressing current trends in facelift procedures, Aston quotes a French writer, Jean-Baptiste Alphonse Karr—the more things change, the more they stay the same [40]. While facelift techniques have undergone a significant evolution since their earliest descriptions, current techniques primarily offer modifications to previously described techniques [41]. Skoog laid much of the foundation for subsequent innovations and refinements in facelift techniques [41,42]. Perhaps one of the most discussed ongoing trends of facial rejuvenation today involves the plane of dissection and manipulation of the SMAS [43]. In recent years, the deep-plane facelift has garnered significant attention and risen in popularity among patients and surgeons alike [44]. While social media marketing seems to imply the novelty of the deep-plane facelift, its origins date back 35 years to Hamra’s original description of his technique [45]. Hamra would critically describe this early iteration of the deep-plane technique as a “short-term improvement but long-term disappointment”, despite its significant advancement and contribution [46]. Innumerable modifications and refinements of the technique would take place thanks to contributions from a variety of surgeons over the next several decades. Jacono is commonly attributed with repopularizing the deep-plane technique, describing an extended deep-plane facelift [47]. Still, many others have published nuances to their own deep-plane techniques [20,30,48,49,50]. Roskies and colleagues describe the experience of six surgeons, including nearly 4000 facelifts, using a limited delamination deep-plane rhytidectomy. This group purports that limited skin undermining with a more lateral deep-plane entry point improves the biomechanics of the composite flap, minimizes dead space, and abbreviates the recovery period [49]. Similarly to this approach, Sadati and associates detail a preservation facelift, which seeks to combine the advantages of the extended deep-plane technique and the high SMAS lift. Preservation refers to the objective of minimizing skin undermining while preserving anatomical boundaries and structures (Figure 1) [50]. Despite growing widespread enthusiasm, even experienced surgeons demand a cautious approach for sub-SMAS dissections [51]. A meta-analysis examining 183 published studies identified sub-SMAS techniques (deep-plane rhytidectomy (OR = 2.22) and high lateral SMAS procedure (OR = 2.71)) as being associated with a higher risk of temporary facial nerve paralysis compared to SMAS plication procedures. However, in this same study, there was no difference identified in permanent facial nerve injury amongst the various techniques [52].

**Figure 2:**
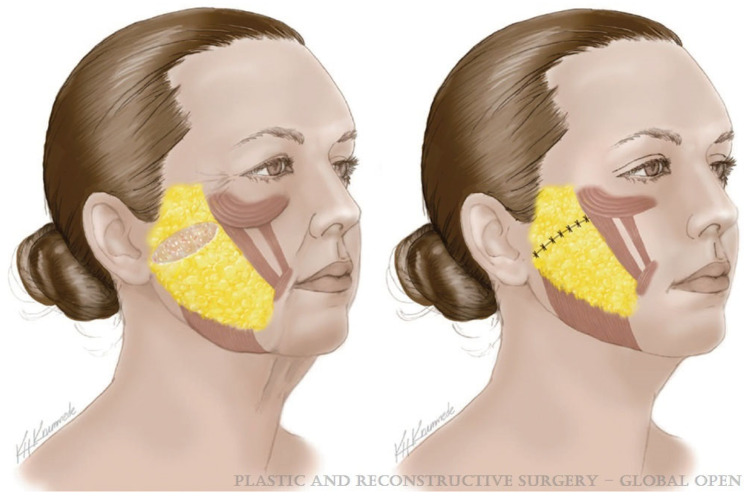
A. Demonstrated is an obliquely oriented SMASectomy that lies perpendicular to the nasolabial fold (**left**). Following SMASectomy, the SMAS is repaired, providing a lift to the facial soft tissues (**right**). This figure was reused with permissions under CC-BY from Wolters Kluwer Health, Inc, License Number 6045420569206. Image is from Figure 11 of Rohrich et al. [59].

SMAS facelift techniques are equally heterogeneous with countless variations and modifications including SMAS plication [53], SMASectomy [54] (Figure 2), high SMAS [55], SMAS stacking [56], and extended SMAS techniques [57]. Avashia and colleagues performed a systematic review of facelift techniques and outcomes, dividing operations into three broad categories—superficial, SMAS manipulation, and SMAS elevation (Table 1) [58]. The authors identified a total of eight overarching techniques and compared aesthetic outcomes as well as complication profiles amongst these. The authors identified comparable aesthetic outcomes between SMAS elevation (deep plane, composite, SMAS flap/extended SMAS, and high SMAS) and SMAS manipulation (SMASectomy, SMAS plication, and SMAS stacking) procedures. There was some, albeit weak, evidence to suggest an increased incidence of complications among SMAS elevation techniques, though there are limited studies reporting direct comparisons between the procedures. Finally, the authors conclude that facial fat grafting remains a central tenet of most modern facelift procedures [58].

Minimally invasive techniques with reduced scar burden will always be included in discussions of facelifting techniques [60]. Ramirez described an endoscopic facelift approach to provide rejuvenation to the central and lower thirds of the face while minimizing the stigmata of surgery [61]. Tonnard’s MACS lift provided another alternative to full pre- and postauricular incisions with a minimal incision technique for facelifting [62]. More recently, Kao detailed an endoscopic deep-plane facelift, coined the ponytail lift, that utilizes incisions concealed in the temple, postauricular, and posterior scalp. Of note, in patients with skin redundancy, a standard posterior auricular incision is required to address the neck skin excess [63]. Mani also describes an endoscopic facelift technique focused on avoiding preauricular incisions while still performing the necklift under direct visualization. The author describes comparable outcomes in patients with the “scarless” and open techniques [64].

**Figure 3:**
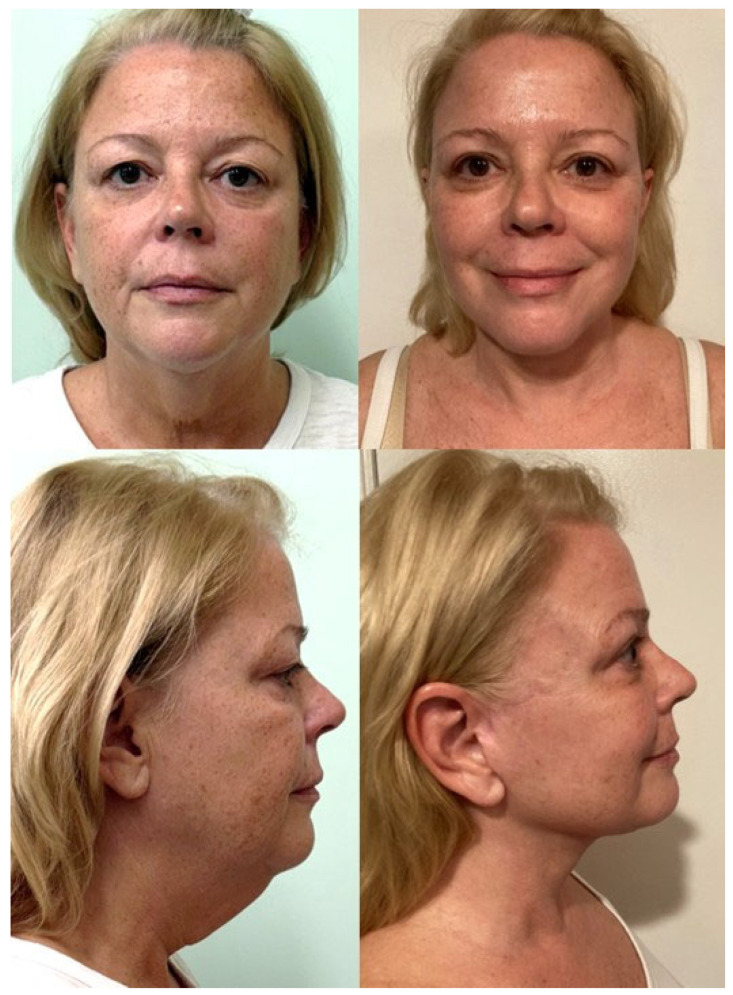
This is a patient of the authors who is shown pre-operatively (**left**) with a heavy neck, an obtuse cervicomental angle, heavy jowling, and an indiscriminate mandibular border. The patient is pictured seven months post-operatively (**right**) from an extended deep-plane facelift with a vertical midline platysmaplasty in combination with superficial neck liposuction. The patient also had a bilateral upper blepharoplasty. The patient has significant improvement in terms of jawline contour, elimination of jowling, and a defined cervicomental angle. Patient provided written consent for the use of images.

Evidence for the superiority of one technique over another is unlikely to be achieved. More important for the surgeon is appropriate knowledge of the benefits and shortcomings of each procedure and adapting the surgical approach based on the presenting patient [58]. Notably, studies examining different facelift techniques performed on identical twins have demonstrated that excellent results can be obtained in similar patients in the hands of an experienced surgeon [65]. Furthermore, Aston described a series of patients receiving different techniques on each side of the face and demonstrated comparable aesthetic outcomes [66]. While debate over which technique provides superior outcomes will persist, more important is understanding the patient’s anatomy and tailoring the operation based on the presenting signs of facial aging unique to that patient [67,68]. It is the strong belief of the authors of this manuscript that there is no singular, universal approach to achieving optimal outcomes. Surgeons should utilize the armamentarium of techniques and ancillary procedures available to provide a customized approach for each patient to achieve a natural, long-lasting result (Figure 3).

### 3.3. Management of the Neck

**Figure 4:**
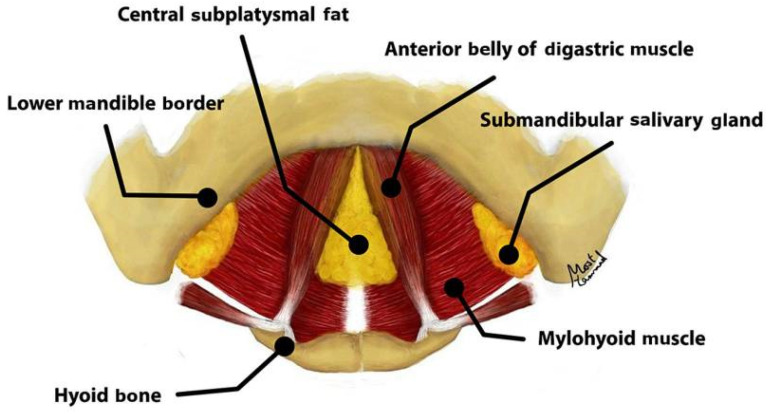
Anatomical depiction of the subplatysmal structures, including the anterior belly of the digastric muscles, subplatysmal fat, and submandibular glands. This figure was reused with permissions under CC-BY from Wolters Kluwer Health, Inc. (Philadelphia, PA, USA), License Number 6018760807739. Image is from Figure 1 of Ghoraba S.M. [71].

Often a difficult problem in facial rejuvenation, improvement in the longevity of neck outcomes and the elimination of recurrent platysmal bands has become an Achilles heel that has spawned many innovations and variations in technique [69]. Management of the neck can be divided into three key areas—the anterior platysma, the subplatysmal structures (subplatysmal fat, digastric muscles, and submandibular glands), and the lateral platysma suspension (Figure 4) [70].

Anterior management of the neck varies significantly based on the patient’s presenting anatomy, amount of subcutaneous adiposity, and degree of plastysmal banding [72]. The importance of platysma manipulation in improving the neck contour has long been acknowledged, despite its challenges with its different decussation patterns and variable viscoelastic properties [73]. Similarly to facelift techniques, innumerable variations in the management of the anterior neck have been reported in the literature. These techniques include subcutaneous elevation in isolation, subcutaneous liposuction, corset platysmaplasty, partial platysmal myotomy, and total platysmal transection [69]. Newer innovations have also been introduced. Recently, Timberlake and colleagues described a midline vertical platysmaplasty, which entails the suspension and advancement of the midline platysmal plication to the mylohyoid fascia with the goal of superiorly repositioning the ptotic platysma by anchoring it to the submental region [30]. Sozer describes a technique of a double-layered midline plication followed by a progressive contouring technique employing barbed sutures to perform vertical suspension of the platysma to the deeper neck and floor of mouth tissue. Suture placement was avoided in the submandibular gland region to avoid marginal mandibular and cervical nerve branches [74].

Jacono has advocated for reduced indications for anterior platysmal tightening procedures, arguing that it limits the vertical lift that can be achieved in the neck from an extended deep-plane facelift technique [75]. He further describes a platysmal hammock, which entails lateral platysmal myotomy along the mandibular border with subsequent suspension of the flap to the mastoid periosteum [76]. Aston questions the novelty of the platysmal hammock and describes that lateral platysma myotomies with subsequent vertical suspension have been performed with several iterations over the past fifty years [40]. Complete platysmal transection in combination with vertical suspension has also been reported as a successful technique for improving the longevity of the effect of the necklift result [77]. Not all authors agree that lateral platysmal myotomy is necessary to improve the jawline and neck contour [30]. Lateral fixation of the platysma has been described by direct tissue apposing sutures, purse-string sutures, and spanning sutures with unclear consequences for the structural integrity and strength of the platysmal suspension [30,50]. Talei and colleagues describe the mastoid crevasse technique as a method of providing more depth to the gonial angle while simultaneously providing stronger fixation of the composite flap. In addition to achieving vertical and horizontal movement in the neck, anchoring deep along the anterior mastoid wall internally rotates the platysmal flap, providing accentuated gonial angle definition [22].

**Figure 5:**
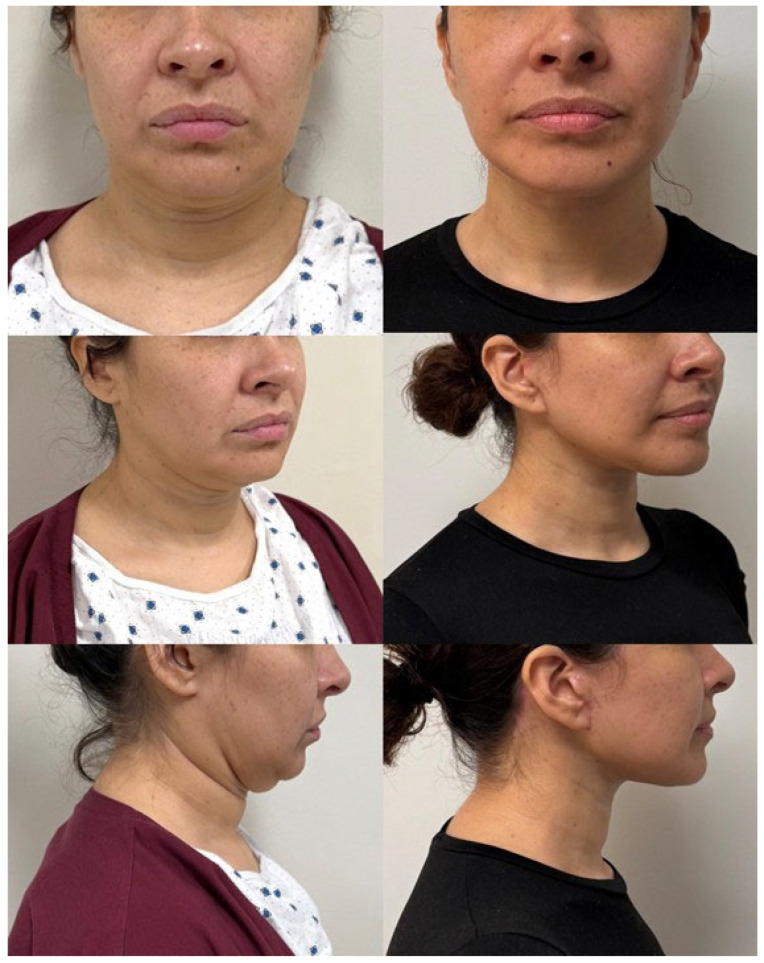
Pre-operative images are demonstrated on the left of a patient of the authors with significant lipodystrophy of the lower face and neck. The patient has jowling and an ill-defined mandibular border. This patient underwent an SMAS plication facelift procedure in conjunction with superficial liposuction of the neck, a vertical anterior platysmaplasty, and lateral subplatysmal flap with suspension along the mastoid fascia. Six-month post-operative photos are visualized on the right. The patient has significant improvement of the jowls and a sharp cervicomental angle without the requirement for any subplatysmal resection. Patient provided written consent for the use of images.

The management of deep neck structures is contested in the literature [78]. Marten affirms that deep neck structures, including subplastysmal fat excess, submandibular gland ptosis or enlargement, and digastric muscle hypertrophy, may contribute to inadequate post-operative neck contour and details pre-operative maneuvers for assessing these structures upon a physical exam (Connell’s view, plastysmal contraction, and palpation of a neck ‘wattle’) [72]. In particular, submandibular gland reduction has become a hot topic in the management of the neck, with as many staunch proponents as dissidents [78]. Those supporting submandibular gland resection assert the aesthetic benefits, with the improvement in jawline definition, devoid of fullness from a ptotic or hypertrophied gland. Opponents warn of potential complications (including significant bleeding, damage to branches of the facial nerve, and concern for sialocele) and an over-sculpted, hollow neck [79]. In hopes of preventing bleeding in the deep neck and reducing the incidence of sialocele, LigaSure (Valleylab, Boulder, CO, USA) has been proposed for submandibular gland excision. Benefits include a reduction in thermal transmission to the surrounding tissue compared to monopolar electrocautery [80]. Others recommend the Harmonic Scalpel (Ethicon, Raritan, NJ, USA) and report that the thermal damage is even less than that of the LigaSure [81]. A systematic review of deep necklift procedures identified 57 studies inclusive of over 8600 patients. The aesthetic outcomes of results were favorable amongst the studies reporting either physician assessment or patient satisfaction. Complications were reported in 60% of studies. The most common complications were neuropraxia (0.2–12%) and hematoma (0.2–4%) with variable incidences [70]. Not included in the prior review, Ghoraba reports a large case series of over 1800 facial rejuvenation patients and performed deep neck surgery in roughly a third (34.4%) of patients with a similar complication profile [71]. While some patients may benefit from deep neck surgery, not all patients require it (Figure 5). Wever highlights the importance of understanding each patient’s unique anatomy, degree and extent of aging in the neck, distribution of soft tissues, underlying bony architecture, skin elasticity, and other factors when determining the appropriate management of the neck for each patient [82].

### 3.4. Other Considerations

While it is not possible to provide an appropriate representation of every aspect of facial rejuvenation in this paper, it must be mentioned that modern facial rejuvenation does not consider face- and necklifts in isolation, but rather encompasses a global perspective including a variety of ancillary surgical and nonsurgical procedures [83,84]. Given the increased elevation of the midface and cheek with modern facelift techniques, concomitant brow-lifting procedures are often needed to avoid soft-tissue crowding and bunching at the lateral canthus [85]. A plethora of techniques have been described, including open and endoscopic approaches [86,87,88,89,90]. Lip lifting and buccal fat reduction have likewise seen a resurgence in popularity as adjuncts to comprehensively treat facial aging and improve facial shape [91,92].

While surgical procedures often yield substantial structural repositioning of the descended facial structures and improve soft-tissue laxity, they fail to address the quality of the skin [93]. To address these limitations, concomitant treatment with an arsenal of different energy devices, including radiofrequency, microneedling, microfocused ultrasound, laser, and combination treatments, has been employed [1,84,94]. Skin resurfacing with non-energy-based treatments includes dermabrasion and chemical peels [1]. Injectable neuromodulators and soft-tissue fillers remain a mainstay of facial rejuvenation for patients both before and after face- and necklift procedures [95]. Rather than simply replacing lost volume with soft-tissue fillers, biostimulators, including platelet-rich plasma (PRP), poly-L-lactic acid, calcium hydroxylapatite, and platelet-rich fibrin, have emerged as potential modalities to increase collagen and alter the physiology of facial aging [96,97]. Innumerable new and old cosmeceutical treatments have demonstrated substantial promise for both pre- and post-procedure treatment [98]. While not an exhaustive list of every treatment available to today’s patient seeking facial rejuvenation, this serves as a reminder that excellent results are often achieved through a combination of various treatments including both surgical and nonsurgical interventions.

Other trends include increased attention to safety during rhytidectomies. Hematomas represent a significant risk for facelift patients, with reported incidences ranging from 1 to 14% of cases [99]. Many studies have characterized patient-level risk factors for hematoma, including high blood pressure (pre- or post-operatively), male sex, and antiplatelet/anticoagulant medications [100,101,102,103]. For many surgeons, hematoma prevention entails a multimodal approach to minimize the risk of bleeding during the peri-operative period. The maintenance of post-operative blood pressure below 120 mmHg has been demonstrated to be beneficial in avoiding hematomas [99]. Compression dressings also remain a mainstay of post-operative management [99]. Post-operative drains have long been used, though they have not been demonstrated to reduce hematoma rates [104,105,106]. Many surgeons have sought to eliminate the need for drains by using alternative methods for managing the dead space from flap dissection. Tissue sealants have been utilized and are a common practice amongst some surgeons for reducing the risk of post-operative bleeding [48,107,108]. The hemostatic net has become a popular option for reducing the risk of hematoma following facelifts. Its proposed benefits are secondary to dead space elimination in the subcutaneous plane [109]. The risk of hyper- or hypopigmentation with the transcutaneous quilting sutures remains the primary limitation of the hemostatic net, though its risk may be mitigated with the use of 5-0 diameter sutures [110]. Over the past several years, tranexamic acid (TXA) has been more commonly integrated into plastic surgery, including in facial rejuvenation [111]. Early data using TXA in combination with local anesthetic for direct infiltration appeared to demonstrate decreased bleeding, shortened operative times, and reduced facelift drainage output [112]. A randomized, controlled, and double-blind study examined the effect of intravenous TXA administration and highlighted a significant reduction in post-operative ecchymosis and fluid collections [113]. A recent study sought to determine the comparative efficacy of TXA administered directly in the tumescent solution versus intravenously. In the metrics assessed (intra-operative bleeding, bruising/swelling, and time to drain removal), the cohort of patients receiving IV TXA demonstrated slightly improved outcomes [114]. While the data surrounding TXA are largely encouraging concerning the reduction in bleeding and swelling, appropriate concerns have been raised with the potential risks of ischemic skin flap-related complications, including skin flap necrosis. Of note, a recent systematic review of TXA use in facelift surgery reports that cases of skin necrosis with the use of TXA have been associated with the local administration of the medication [115].

In hopes of reducing the length of recovery, facelift procedures performed under total local anesthesia have risen in popularity [116]. Given the number of ancillary facial rejuvenation procedures that can be readily performed on a same-day, wide-awake basis, patients have pushed surgeons towards performing facelift procedures without the requirement for general anesthesia [116,117]. A large case series suggests that the benefits extend beyond the avoidance of general anesthesia, but suggests that a facelift performed under local anesthesia may reduce the incidence of hematoma by maintaining a narrower window of blood pressure both during and after the procedure [118]. As is the case for all aspects of facial rejuvenation, patients and surgeons should work together to determine the optimal approach for each patient.

### 3.5. Limitations and Future Directions

While many individual techniques have been described for facelift and necklift procedures, high-level evidence comparing aesthetic outcomes and complication profiles is limited. As these procedures are invariably surgeon-dependent, adequate comparisons amongst techniques will remain a challenge. The objective of this paper was to sample the diversity of the recent literature related to current trends in facial rejuvenation with a special focus on facelift and necklift procedures. This manuscript was a narrative review, and analysis of the full breadth of the available literature was outside the scope of this study. As such, the study did not have strict inclusion or exclusion criteria consistent with higher-level review papers, such as a systematic review or meta-analysis. Because of this, the analysis presented should be interpreted in this context. Surgeons should be encouraged, when possible, to report outcome metrics such as the FACE-Q to provide objective data on aesthetic outcomes [119]. Doing so will help substantiate the available literature and continually advance both patient safety and aesthetic outcomes.

## 4. Conclusions

Facelift and necklift procedures remain the foundational pillars for facial rejuvenation. Plastic surgeons have given renewed attention to the soft-tissue anatomy of the face and neck to optimize surgical procedures and reduce complications. Ongoing debates will persist regarding the overall efficacy and longevity of the different surgical techniques, though the literature demonstrates that excellent results can be achieved with a variety of techniques. This accentuates the importance of understanding the needs of the patient presenting for facial rejuvenation and tailoring the operation based on patient-specific anatomy. Other surgical procedures and non-surgical treatments should be considered to optimize and enhance facelift and necklift results.

## Figures and Tables

**Table 1:** Facelift technique classification based on the level of dissection and manipulation of the superficial musculoaponerutotic system (SMAS). Adapted from Avashia et al., 2022 [58].

Technique	Examples
Superficial	Subcutaneous
SMAS manipulation	SMASectomy SMAS plication SMAS stacking
SMAS elevation	High SMAS SMAS flap/extended SMAS Deep plane Composite
